# The role of inflammation in neurological disorders: a brief overview of multiple sclerosis, Alzheimer’s, and Parkinson’s disease’

**DOI:** 10.3389/fneur.2024.1439125

**Published:** 2024-10-29

**Authors:** Yahveth Cantero-Fortiz, Mercè Boada

**Affiliations:** ^1^Ace Alzheimer Center Barcelona, Universitat Internacional de Catalunya, Barcelona, Spain; ^2^Networking Research Center on Neurodegenerative Diseases (CIBERNED), Instituto de Salud Carlos III, Madrid, Spain

**Keywords:** central nervous system, Alzheimer’s disease, multiple sclerosis, Parkinson’s disease, neuroinflammation, biomarkers

## Abstract

Neuroinflammation is a central feature in the pathophysiology of several neurodegenerative diseases, including MS, AD, and PD. This review aims to synthesize current research on the role of inflammation in these conditions, emphasizing the potential of inflammatory biomarkers for diagnosis and treatment. We highlight recent findings on the mechanisms of neuroinflammation, the utility of biomarkers in disease differentiation, and the implications for therapeutic strategies. Advances in understanding inflammatory pathways offer promising avenues for developing targeted interventions to improve patient outcomes. Future research should focus on validating these biomarkers in larger cohorts and integrating them into clinical practice to enhance diagnostic accuracy and therapeutic efficacy.

## Introduction

Neurodegenerative diseases, including multiple sclerosis (MS), Alzheimer’s disease (AD), and Parkinson’s disease (PD), remain among the most debilitating challenges in modern medicine, characterized by progressive neuronal loss and dysfunction that lead to severe cognitive, motor, and behavioral impairments. While it is well-established that inflammation within the central nervous system (CNS) plays a critical role in the pathogenesis and progression of these disorders, the exact nature of this inflammatory response—and whether it manifests uniformly across different diseases—remains a subject of significant debate.

Current research suggests that inflammation is not a monolithic process but rather exhibits disease-specific characteristics, raising questions about whether a one-size-fits-all approach can be effective in managing neurodegenerative conditions. For instance, while MS is marked by autoimmune-mediated demyelination (1–3), AD and PD involve neuroinflammation driven by proteinopathies such as amyloid-beta plaques, tau tangles, and α-synuclein aggregates (4–9). These differences in the underlying mechanisms of inflammation across these diseases suggest that the therapeutic strategies needed to target neuroinflammation effectively may also need to be tailored accordingly.

This review will explore the complex and varied roles of neuroinflammation in MS, AD, and PD, focusing on how these differences impact disease progression and therapeutic outcomes. We will critically examine the utility of inflammatory biomarkers, such as C-reactive protein (CRP), IL-6, and tumor necrosis factor-alpha (TNF-α), in diagnosing and differentiating these conditions (10, 11), as well as their potential for guiding the development of targeted therapies. By highlighting the gaps and controversies in the current understanding of neuroinflammation, this review aims to provide new insights into the pathophysiology of neurodegenerative diseases and contribute to the ongoing debate on how best to treat these challenging disorders.

### Multiple sclerosis

MS is a complex autoimmune disorder marked by chronic inflammation, demyelination, and neurodegeneration within the CNS. While the roles of pro-inflammatory cytokines such as interleukin-1 beta (IL-1β), TNF-α, and interferon-gamma (IFN-*γ*) in disrupting the blood–brain barrier (BBB) and facilitating immune cell infiltration are well documented (1, 2). Recent advances in Large-scale genome-wide association studies (GWAS) have identified genetic variants, such as those in IL7R and IL2RA, which influence immune response and inflammation (12). These insights are shaping new therapeutic strategies that target specific immune pathways.

One significant advancement is the development of B cell-depleting therapies, such as ocrelizumab, which have shown promise in reducing disease activity by targeting CD20-positive B cells and thereby diminishing the production of autoantibodies and pro-inflammatory cytokines (3). Additionally, Janus kinase (JAK) inhibitors, which interfere with cytokine signaling pathways, are emerging as potential treatments that can modulate immune responses more precisely and reduce the progression of neurodegeneration (4). These therapies represent a shift toward more targeted interventions, addressing the underlying immune dysregulation in MS.

In parallel, the role of biomarkers in MS has garnered significant attention, particularly neurofilament light chain (NfL), which serves as a marker for neuroaxonal damage. NfL levels correlate with disease activity and have been increasingly used to monitor treatment efficacy and disease progression (5, 6, 13, 14). However, recent findings highlight some limitations of NfL, such as its lack of specificity for MS compared to other neurodegenerative conditions, prompting ongoing research into complementary biomarkers that could offer more precise insights into disease mechanisms (7–9, 14).

Overall, while traditional approaches have focused on broad immunosuppression, these recent developments in targeted therapies and biomarker utilization are paving the way for more personalized and effective management of MS.

#### Alzheimer’s disease

AD is the most common cause of dementia, characterized by amyloid-beta plaques, tau tangles, and significant neuroinflammation. Peripheral inflammation, as indicated by biomarkers such as the neutrophil-to-lymphocyte ratio (NLR) and CRP, correlates with disease severity and has been linked to an increased risk and faster progression of AD (5, 10). Microglial activation plays a pivotal role in AD-related neuroinflammation, with activated microglia producing cytokines and chemokines (IL-6, TNF- *α*) that perpetuate neuronal damage and promote amyloid-beta plaque formation (10). Chronic inflammatory diseases such as rheumatoid arthritis and psoriasis are associated with an increased risk of AD, suggesting that systemic inflammation may exacerbate AD pathology (11). Additionally, genetic studies have revealed variants in genes like APOE and TREM2, which are heavily implicated in modulating the inflammatory response, particularly through microglial activation (15, 16). Recent advancements in understanding AD have highlighted the role of toll-like receptors (TLRs) in recognizing amyloid-beta and triggering inflammatory responses, underscoring the complex interplay between innate immunity and neurodegeneration (17, 18). Monoclonal antibodies targeting amyloid-beta and tau proteins have emerged as promising therapeutic candidates. Clinical trials of these antibodies, such as Aducanumab, Lecanemab, and Donanemab, have demonstrated their ability to reduce amyloid burden in the brain, offering hope for modifying the course of the disease (19, 20). However, these therapies have also faced significant challenges, including mixed results in terms of clinical efficacy and concerns over adverse effects such as amyloid-related imaging abnormalities (ARIA). These findings have spurred ongoing research into optimizing dosing regimens, patient selection, and combination therapies that might enhance the benefits while minimizing risks (21).

The success and limitations of these monoclonal antibodies in recent trials highlight both the potential and the complexity of developing effective AD therapies. As the field continues to evolve, these insights will be crucial in guiding the future of AD treatment, particularly in the pursuit of therapies that not only reduce amyloid and tau pathologies but also address the broader inflammatory processes that contribute to neurodegeneration (22).

#### Parkinson’s disease

PD is a neurodegenerative disorder characterized by motor dysfunction and the presence of Lewy bodies. Neuroinflammation, involving both the innate and adaptive immune systems, is a hallmark of PD. Inflammatory biomarkers in CSF can distinguish PD from other neurodegenerative diseases, such as AD and Dementia with Lewy Body (DLB), offering the potential for early diagnosis and targeted treatment strategies (23).

In PD, α-synuclein aggregates trigger an inflammatory response mediated by microglia and astrocytes, which produce pro-inflammatory cytokines such as TNF-α, IL-1β, and IL-6 (6, 7). These cytokines are elevated in the brains and CSF of PD patients and contribute to the degeneration of dopaminergic neurons in the substantia nigra, leading to the characteristic motor symptoms of PD (6–9, 23, 24). Recent GWAS findings have identified several immune-related gene variants, such as LRRK2, that modulate the inflammatory response in PD. Understanding these genetic influences could enhance personalized treatment approaches (6–8, 25, 26).

Recent research has underscored the importance of the gut-brain axis in PD, particularly the role of the gut microbiota in modulating neuroinflammation. Alterations in the gut microbiota composition have been linked to PD, where certain microbial profiles may promote a pro-inflammatory state that exacerbates neurodegenerative processes. For example, studies have shown that the abundance of certain bacterial strains correlates with elevated levels of pro-inflammatory cytokines in PD patients, suggesting a bidirectional relationship between gut dysbiosis and neuroinflammation (27). This has led to the exploration of therapies aimed at modulating the gut microbiota as a novel approach to influencing neuroinflammatory pathways in PD.

The use of anti-inflammatory drugs in PD has been an area of active investigation, though with mixed results. Glucocorticoids and nonsteroidal anti-inflammatory drugs (NSAIDs) have been tested in various clinical trials, with some showing modest effects in reducing neuroinflammation and slowing disease progression, while others have failed to demonstrate significant clinical benefits (25, 26, 28). These outcomes highlight the challenges in targeting neuroinflammation in PD and suggest that future therapies may need to combine anti-inflammatory strategies with other approaches, such as neuroprotection and microbiota modulation. As research continues to evolve, understanding the complex interplay between the immune system, genetics, and microbiota will be crucial in developing more effective treatments for PD. However, the failure of anti-inflammatory drugs like NSAIDs and glucocorticoids in clinical trials highlights the complexity of targeting neuroinflammation in PD. Future therapies may need to combine these with other strategies, such as neuroprotection and microbiota modulations (29).

#### Mechanistic similarities and differences in inflammation in MS, AD, and PD

These diseases share common inflammatory pathways including the activation of microglia and astrocytes. Cytokines (TNF-α, IL-1β, and IL-6) perpetuate neuronal dysfunction and cell death, contributing to the progression of neurodegeneration. Additionally, BBB is compromised in each disease, allowing peripheral immune cells to enter the brain, which exacerbates the inflammatory response. This disruption of the BBB, along with the sustained production of inflammatory cytokines, represents a shared inflammatory profile across these neurodegenerative diseases (2, 4, 10, 30–32).

Despite these similarities, the underlying genetic and inflammatory mechanisms differ substantially. In MS, the inflammatory response is primarily driven by T and B lymphocytes, resulting in an autoimmune attack against the myelin in the CNS, which justifies the use of therapies like ocrelizumab that deplete B cells. In AD, the inflammation is triggered by the accumulation of misfolded proteins, such as beta-amyloid plaques and tau tangles, which activate microglia through TLRs. This protein-driven inflammation distinguishes AD from other diseases. Meanwhile, in PD, inflammation is linked to the accumulation of α-synuclein aggregates in dopaminergic neurons, which activate microglia and contribute to neuronal degeneration (1, 16, 25, 30). This mechanistic divergence suggests that treatment approaches must be disease-specific rather than generalized across all neurodegenerative disorders.

#### Inflammatory biomarkers and diagnostic potential

The identification of specific inflammatory biomarkers holds great promise for improving the accuracy of diagnosis and the development of targeted therapies for neurodegenerative diseases. Panels of biomarkers, such as those identified for PD, have shown potential in differentiating it from AD and DLB, which is crucial for early intervention and personalized treatment approaches (11). These inflammatory markers—including NfL, glial fibrillary acidic protein (GFAP), CRP, IL-6, and TNF-α—can be measured in CSF and blood, providing non-invasive tools for clinical practice (1, 6, 10, 17, 18, 24, 33).

Recent advances have introduced several emerging biomarkers that offer disease-specific insights, enhancing our ability to tailor treatments to individual patients. For example, plasma levels of phosphorylated tau (p-tau) in AD have gained attention as a promising biomarker that correlates strongly with tau pathology and cognitive decline, offering an advantage over traditional CSF biomarkers (34). In PD, α-synuclein seed amplification assays are emerging as a highly specific tool for detecting misfolded α-synuclein, potentially distinguishing PD from other synucleinopathies with greater accuracy (21). Additionally, in DLB, biomarkers such as DJ-1 protein and cerebrospinal fluid neurogranin have been identified, offering further refinement in differentiating DLB from both AD and PD (35).

Techniques such as the proximity extension assay (PEA) enable multiplex analysis of these emerging and established inflammatory CSF biomarkers, providing deeper insights into disease mechanisms and progression for PD and AD (23). As these biomarker panels evolve, they offer the potential for developing personalized treatment plans tailored to the specific inflammatory profiles of individual patients.

However, the utility of these biomarkers in routine clinical practice remains limited by their variability across patients and lack of standardization. Additionally, longitudinal studies are necessary to validate these markers and understand their progression-related dynamics. Ongoing identification and validation of emerging biomarkers are key to advancing personalized medicine in neurodegenerative diseases (36).

#### Therapeutic implications

Targeting specific inflammatory pathways offers a promising avenue for developing personalized, since therapies for neurodegenerative diseases the variability in patient response to treatments underscores the importance of identifying individual inflammatory profiles and tailoring interventions accordingly. This approach is central to advancing personalized medicine, where therapies can be adapted to the unique biological characteristics of each patient (37).

Biological agents targeting specific cytokines or immune pathways are at the forefront of this personalized approach. In particular, recent advancements in monoclonal antibodies targeting amyloid-beta and tau proteins in AD have shown promise in clinical trials. For example, aducanumab and lecanemab have demonstrated the ability to reduce amyloid burden in the brain, which could slow disease progression. These therapies, while promising, also face challenges, highlighting the need for careful patient selection and monitoring in clinical practice (16, 38, 39).

For MS, B-cell depleting therapies, like ocrelizumab, have significantly advanced the treatment landscape by targeting CD20-positive B cells in the autoimmune response that characterizes MS. Ocrelizumab has demonstrated efficacy in reducing disease activity and slowing progression, particularly in relapsing forms of MS. These outcomes suggest that targeting specific immune cell populations can be an effective strategy in managing neuroinflammation and its associated neurodegeneration in MS (40).

In PD, antibodies such as prasinezumab are designed to target these α-synuclein aggregates, aiming to reduce their neurotoxic effects. Although still in the early stages, these therapies hold promise for slowing the progression of PD by directly addressing one of the underlying mechanisms of neurodegeneration (41).

In addition to pharmacological interventions, lifestyle modifications play a crucial role in modulating inflammatory responses. Dietary changes, physical exercise, and stress reduction have been shown to complement pharmacological treatments by reducing systemic and CNS inflammation (42, 43). Furthermore, the emerging understanding of the gut-brain axis and the role of gut microbiota in regulating inflammation has opened new therapeutic avenues. Probiotics and prebiotics are being explored for their potential to influence neuroinflammatory pathways, offering another layer of personalized treatment options (44, 45).

## Discussion

Inflammation’s role in neurodegenerative diseases is increasingly recognized as a critical factor in their pathogenesis and progression. While the studies reviewed demonstrate the significance of inflammatory biomarkers in diagnosing and differentiating between MS, AD, and PD, the translation of these findings into clinical practice remains challenging. One of the major obstacles is the overlapping inflammatory mechanisms across these diseases, such as the activation of immune cells, BBB breakdown, and production of pro-inflammatory cytokines like IL-1β, TNF-α, and IL-6, all of which contribute to neuronal damage and disease progression (1–9). This overlap complicates the development of disease-specific biomarkers and targeted therapies.

Also, the interplay between systemic and CNS inflammation further complicates the clinical landscape. While systemic markers such as CRP and NLR are elevated in neurodegenerative conditions their direct relationship with CNS pathology and disease progression remains poorly understood (4–6). This raises questions about the specificity and utility of these markers in routine clinical diagnostics.

Emerging therapeutic strategies targeting neuroinflammation show promise, but significant challenges remain. The variability in patient responses to treatments such as monoclonal antibodies and cytokine inhibitors underscores the need for personalized approaches. This variability is influenced by factors such as genetic predisposition, disease stage, and comorbidities, highlighting the limitations of current therapeutic strategies (46). Additionally, age and sex are increasingly recognized as important modulators of inflammatory responses, affecting the onset, progression, and severity of neurodegenerative diseases, as well as the efficacy of therapeutic interventions (30).

In parallel, lifestyle modifications—such as diet, exercise, and stress management—have shown efficacy in modulating both systemic and CNS inflammation. Incorporating these interventions into personalized treatment plans, tailored to the patient’s specific inflammatory profile and overall health status, may offer a more comprehensive approach to managing neuroinflammation, ultimately improving clinical outcomes (47).

As our understanding of these factors deepens, one critical area that warrants further exploration is the genetic modulation of inflammation in neurodegenerative diseases. GWAS have identified several genetic risk factors linked to immune response in AD, PD and MS. For AD, in addition to well-known genes like APOE and TREM2, GWAS has uncovered additional genetic variants associated with immune regulation, many of which are specifically expressed in microglia, such as MS4As, CD33, SPI1, and INPP5D, or are enriched in microglial cells, like CR1, ABCA7, and CLU. These findings suggest that microglia play a crucial role in the immune regulation of AD. However, further studies are needed to verify the role of these molecules in the development and progression of AD (15, 16).

Similarly, in PD, GWAS has identified several immune-related gene variants, including LRRK2, GBA, PRKN, and PINK1. These genetic factors have been linked to altered inflammatory responses, which may contribute to disease susceptibility and progression in PD. The identification of common genetic variants between PD and other inflammatory diseases further underscores the importance of understanding the genetic underpinnings of inflammation in neurodegenerative conditions (15, 26).

For MS, GWAS has identified variants in IL7R and IL2RA, highlighting the immune system’s role. Subsequent studies confirmed these and identified 29 additional risk variants, focusing on genes involved in lymphocyte function, Vitamin D metabolism (CYP27B1 and CYP24A1), and targets of MS immune-modulatory therapies (VCAM1 and IL2RA) (12).

Clarifying how genetic profiles interact with environmental factors and inflammatory processes is key to enhancing our understanding of neurodegenerative disease mechanisms. However, current research is hindered by small cohort sizes and the lack of longitudinal data, limiting our capacity to fully grasp the complexity of genetic contributions to neuroinflammation.

### Future directions

Future research should validate inflammatory biomarkers in larger, diverse cohorts to confirm their diagnostic and therapeutic value. Exploring the genetic basis of inflammation in neurodegenerative diseases is also crucial, with recent studies linking TREM2 variants to altered microglial function in AD and HLA genes to MS susceptibility (15, 16, 48). Additionally, investigating genetic and environmental modulation of inflammation, alongside advanced imaging and biomarker analysis, could deepen understanding and improve monitoring of disease progression. Integrating multi-omics with clinical data may reveal novel therapeutic targets, essential for advancing the field and enhancing patient outcomes (49–51).

## Conclusion

By leveraging recent insights into immune mechanisms, such as microglial activation in AD and genetic influences in MS and PD, the field is advancing toward more personalized approaches in treatment. Rather than simply managing symptoms, these strategies aim to target the specific inflammatory pathways unique to each patient. To make this a reality, future research must focus on precisely modulating immune responses according to individual genetic and environmental profiles. As these methods evolve, the potential to slow disease progression and improve patient outcomes becomes increasingly clear.

Furthermore, the integration of personalized and precision medicine, particularly through the use of inflammatory biomarkers, will be essential. Future efforts should prioritize refining these biomarkers while ensuring their implementation in clinical practice, where they could substantially enhance diagnostic accuracy and therapeutic effectiveness. Accelerating this transition through large-scale clinical trials and collaborative research initiatives will be key to addressing the pressing need for tailored therapies in MS, AD, and PD (52).

The time has come for a concerted effort to move these scientific advancements into clinical settings, paving the way for more effective, individualized interventions that can transform the treatment landscape of neurodegenerative diseases.

## References

[1] Bar-Or A Oliveira EM Anderson DE Hafler DA. Molecular pathogenesis of multiple sclerosis. J Neuroimmunol. (1999) 100:252–9. doi: 10.1016/s0165-5728(99)00193-9 10695735

[2] da Fonseca AC Matias D Garcia C Amaral R Geraldo LH Freitas C. The impact of microglial activation on blood-brain barrier in brain diseases. Front Cell Neurosci. (2014) 8:362. doi: 10.3389/fncel.2014.00362 25404894 PMC4217497

[3] Stys PK Zamponi GW van Minnen J Geurts JJG. Will the real multiple sclerosis please stand up? Nat Rev Neurosci. (2012) 13:507–14. doi: 10.1038/nrn3275, PMID: 22714021

[4] Heneka MT Golenbock DT Latz E. Innate immunity in Alzheimer's disease. Nat Immunol. (2015) 16:229–36. doi: 10.1038/ni.3102 25689443

[5] Kara SP Altunan B Ünal A. Investigation of the peripheral inflammation (neutrophil-lymphocyte ratio) in two neurodegenerative diseases of the central nervous system. Neurol Sci. (2021) 43:1799–807. doi: 10.1007/s10072-021-05507-5 34331157 PMC8324446

[6] Mogi M Harada M Kondo T Riederer P Nagatsu T. Brain-derived interleukin-6 in patients with Parkinson's disease. Neurosci Lett. (1994) 165:208–10. doi: 10.1016/0304-3940(94)90746-3 8015728

[7] Lema Tomé CM Tyson T Rey NL Grathwohl S Britschgi M Brundin P. Inflammation and α-synuclein’s prion-like behavior in Parkinson’s disease--is there a link?. Mol Neurobiol. (2013) 47:561–74. doi: 10.1007/s12035-012-8267-8 22544647 PMC3589652

[8] Moehle MS West AB. M1 and M2 immune activation in Parkinson's disease: foe and ally? Neuroscience. (2015) 302:59–73. doi: 10.1016/j.neuroscience.2014.11.018, PMID: 25463515 PMC4442748

[9] Dzamko N Gysbers A Bandopadhyay R Bolliger MF Uchino A Zhao Y. LRRK2 levels and phosphorylation in Parkinson’s disease brain and cases with restricted Lewy bodies. Mov Disord. (2017) 32:423–432. doi: 10.1002/mds.26892 27911006

[10] Holmes C Cunningham C Zotova E Culliford D Perry VH Cox P. Systemic inflammation and disease progression in Alzheimer disease. Neurology. (2009) 73:768–74. doi: 10.1212/WNL.0b013e3181b6bb95, PMID: 19738171 PMC2848584

[11] Novoa C Salazar P Cisternas P Gherardelli C Vera-Salazar R Zolezzi JM. Inflammation context in Alzheimer's disease, a relationship intricate to define. Biol Res. (2022) 55:39. doi: 10.1186/s40659-022-00404-3 36550479 PMC9784299

[12] Ferrè L Filippi M Esposito F. Involvement of genetic factors in multiple sclerosis. Front Cell Neurosci. (2020) 14:612953. doi: 10.3389/fncel.2020.612953, PMID: 33335478 PMC7735985

[13] Ning L Wang B. Neurofilament light chain in blood as a diagnostic and predictive biomarker for multiple sclerosis: a systematic review and meta-analysis. PLoS One. (2022) 17:e0274565. doi: 10.1371/journal.pone.0274565, PMID: 36103562 PMC9473405

[14] Kouchaki E Dashti F Mirazimi SMA Alirezaei Z Jafari SH Hamblin MR. Neurofilament light chain as a biomarker for diagnosis of multiple sclerosis. EXCLI J. (2021) 20:1308–25. doi: 10.17179/excli2021-3973 34602928 PMC8481790

[15] Zhang W Xiao D Mao Q Xia H. Role of neuroinflammation in neurodegeneration development. Signal Transduct Target Ther. (2023) 8:267. doi: 10.1038/s41392-023-01486-5, PMID: 37433768 PMC10336149

[16] Fortea J Pegueroles J Alcolea D Belbin O Dols-Icardo O Vaqué-Alcázar L. Publisher correction: APOE4 homozygosity represents a distinct genetic form of Alzheimer's disease. Nat Med. (2024) 30:2093. doi: 10.1038/s41591-024-03127-y 38886628

[17] Hodgson L Li Y Iturria-Medina Y Stratton JA Wolf G Krishnaswamy S. Supervised latent factor modeling isolates cell-type-specific transcriptomic modules that underlie Alzheimer's disease progression. Commun Biol. (2024) 7:591. doi: 10.1038/s42003-024-06273-8, PMID: 38760483 PMC11101463

[18] Heppner FL Ransohoff RM Becher B. Immune attack: the role of inflammation in Alzheimer disease. Nat Rev Neurosci. (2015) 16:358–72. doi: 10.1038/nrn3880, PMID: 25991443

[19] Sevigny J Chiao P Bussière T Weinreb PH Williams L Maier M. The antibody aducanumab reduces Aβ plaques in Alzheimer’s disease. Nature. (2016) 537:50–6. doi: 10.1038/nature19323 27582220

[20] Cummings J. Anti-amyloid monoclonal antibodies are transformative treatments that redefine Alzheimer's disease therapeutics. Drugs. (2023) 83:569–76. doi: 10.1007/s40265-023-01858-9, PMID: 37060386 PMC10195708

[21] Doran SJ Sawyer RP. Risk factors in developing amyloid related imaging abnormalities (ARIA) and clinical implications. Front Neurosci. (2024) 18:1326784. doi: 10.3389/fnins.2024.1326784 38312931 PMC10834650

[22] Neațu M Covaliu A Ioniță I Jugurt A Davidescu EI Popescu BO. Monoclonal antibody therapy in Alzheimer's disease. Pharmaceutics. (2023) 16:60. doi: 10.3390/pharmaceutics16010060 38258071 PMC11154277

[23] Pedersen CC Ushakova AY Skogseth R Alves G Tysnes OB Aarsland D. Inflammatory biomarkers in newly diagnosed patients with Parkinson disease and related neurodegenerative disorders. Neurol Neuroimmunol Neuroinflamm. (2023) 10:e200132. doi: 10.1212/NXI.0000000000200132 37258413 PMC10231912

[24] Tansey MG Goldberg MS. Neuroinflammation in Parkinson's disease: its role in neuronal death and implications for therapeutic intervention. Neurobiol Dis. (2010) 37:510–8. doi: 10.1016/j.nbd.2009.11.004, PMID: 19913097 PMC2823829

[25] Hirsch EC Hunot S. Neuroinflammation in Parkinson's disease: a target for neuroprotection? Lancet Neurol. (2009) 8:382–97. doi: 10.1016/S1474-4422(09)70062-6 19296921

[26] Tansey MG Wallings RL Houser MC Herrick MK Keating CE Joers V. Inflammation and immune dysfunction in Parkinson disease. Nat Rev Immunol. (2022) 22:657–73. doi: 10.1038/s41577-022-00684-6, PMID: 35246670 PMC8895080

[27] Mitrea L Nemeş SA Szabo K Teleky BE Vodnar DC. Guts imbalance imbalances the brain: a review of gut microbiota association with neurological and psychiatric disorders. Front Med. (2022) 9:813204. doi: 10.3389/fmed.2022.813204, PMID: 35433746 PMC9009523

[28] Gagne JJ Power MC. Anti-inflammatory drugs and risk of Parkinson disease: a meta-analysis. Neurology. (2010) 74:995–1002. doi: 10.1212/WNL.0b013e3181d5a4a3, PMID: 20308684 PMC2848103

[29] Cossu D Hatano T Hattori N. The role of immune dysfunction in Parkinson’s disease development. Int J Mol Sci. (2023) 24:16766. doi: 10.3390/ijms242316766, PMID: 38069088 PMC10706591

[30] Childs R Karamacoska D Lim CK Steiner-Lim GZ. "Let's talk about sex, inflammaging, and cognition, baby": a meta-analysis and meta-regression of 106 case-control studies on mild cognitive impairment and Alzheimer's disease. Brain Behav Immun Health. (2024) 40:100819. doi: 10.1016/j.bbih.2024.100819, PMID: 39161876 PMC11331696

[31] Nazeri M Bazrafshan H Abolhasani FA. Serum inflammatory markers in patients with multiple sclerosis and their association with clinical manifestations and MRI findings. Acta Neurol Belg. (2022) 122:1187–93. doi: 10.1007/s13760-021-01647-9, PMID: 33837496

[32] Qu Y Li J Qin Q Wang D Zhao J An K. A systematic review and meta-analysis of inflammatory biomarkers in Parkinson's disease. NPJ Parkinsons Dis. (2023) 9:18. doi: 10.1038/s41531-023-00449-5, PMID: 36739284 PMC9899271

[33] Teunissen CE Otto M Engelborghs S Herukka SK Lehmann S Lewczuk P. A consensus protocol for the standardization of cerebrospinal fluid collection and biobanking. Neurology. (2009) 73:1914–22. doi: 10.1212/WNL.0b013e3181c47cc2 19949037 PMC2839806

[34] Gonzalez-Ortiz F Kac PR Brum WS Zetterberg H Blennow K Karikari TK. Plasma phospho-tau in Alzheimer's disease: towards diagnostic and therapeutic trial applications. Mol Neurodegener. (2023) 18:18. doi: 10.1186/s13024-023-00605-8, PMID: 36927491 PMC10022272

[35] Abdelmoaty MM Lu E Kadry R Foster EG Bhattarai S Mosley RL. Clinical biomarkers for Lewy body diseases. Cell Biosci. (2023) 13:209. doi: 10.1186/s13578-023-01152-x, PMID: 37964309 PMC10644566

[36] Agnello L Gambino CM Ciaccio AM Masucci A Vassallo R Tamburello M. Molecular biomarkers of neurodegenerative disorders: a practical guide to their appropriate use and interpretation in clinical practice. Int J Mol Sci. (2024) 25:4323. doi: 10.3390/ijms25084323, PMID: 38673907 PMC11049959

[37] Griñán-Ferré C Bellver-Sanchis A Guerrero A Pallàs M. Advancing personalized medicine in neurodegenerative diseases: the role of epigenetics and pharmacogenomics in pharmacotherapy. Pharmacol Res. (2024) 205:107247. doi: 10.1016/j.phrs.2024.107247, PMID: 38834164

[38] Rahman A Hossen MA Chowdhury MFI Bari S Tamanna N Sultana SS. Aducanumab for the treatment of Alzheimer's disease: a systematic review. Psychogeriatrics. (2023) 23:512–22. doi: 10.1111/psyg.12944, PMID: 36775284 PMC11578022

[39] van Dyck CH Swanson CJ Aisen P Bateman RJ Chen C Gee M. Lecanemab in early Alzheimer's disease. N Engl J Med. (2023) 388:9–21. doi: 10.1056/NEJMoa2212948 36449413

[40] Lamb YN. Ocrelizumab: a review in multiple sclerosis. Drugs. (2022) 82:323–34. doi: 10.1007/s40265-022-01672-9, PMID: 35192158 PMC8862399

[41] Jamal F. Immunotherapies targeting α-Synuclein in Parkinson disease. Fed Pract. (2020) 37:375–9. doi: 10.12788/fp.0026, PMID: 32908345 PMC7473734

[42] Villeda SA Luo J Mosher KI Zou B Britschgi M Bieri G. The ageing systemic milieu negatively regulates neurogenesis and cognitive function. Nature. (2011) 477:90–4. doi: 10.1038/nature10357 21886162 PMC3170097

[43] Gleeson M Bishop NC Stensel DJ Lindley MR Mastana SS Nimmo MA. The anti-inflammatory effects of exercise: mechanisms and implications for the prevention and treatment of disease. Nat Rev Immunol. (2011) 11:607–15. doi: 10.1038/nri3041, PMID: 21818123

[44] Sampson TR Debelius JW Thron T Janssen S Shastri GG Ilhan ZE. Gut microbiota regulate motor deficits and neuroinflammation in a model of Parkinson’s disease. Cell. (2016) 167:1469–1480.e12. doi: 10.1016/j.cell.2016.11.018, PMID: 27912057 PMC5718049

[45] Sharon G Sampson TR Geschwind DH Mazmanian SK. The central nervous system and the gut microbiome. Cell. (2016) 167:915–32. doi: 10.1016/j.cell.2016.10.027, PMID: 27814521 PMC5127403

[46] Singh K Sethi P Datta S Chaudhary JS Kumar S Jain D. Advances in gene therapy approaches targeting neuroinflammation in neurodegenerative diseases. Ageing Res Rev. (2024) 98:102321. doi: 10.1016/j.arr.2024.102321, PMID: 38723752

[47] Kip E Parr-Brownlie LC. Healthy lifestyles and wellbeing reduce neuroinflammation and prevent neurodegenerative and psychiatric disorders. Front Neurosci. (2023) 17:1092537. doi: 10.3389/fnins.2023.1092537, PMID: 36875655 PMC9975355

[48] Maghbooli Z Sahraian MA Naser MA. Multiple sclerosis and human leukocyte antigen genotypes: focus on the Middle East and North Africa region. Mult Scler J Exp Transl Clin. (2020) 6:2055217319881775. doi: 10.1177/2055217319881775 31976083 PMC6956601

[49] Lorefice L Pitzalis M Murgia F Fenu G Atzori L Cocco E. Omics approaches to understanding the efficacy and safety of disease-modifying treatments in multiple sclerosis. Front Genet. (2023) 14:1076421. doi: 10.3389/fgene.2023.1076421, PMID: 36793897 PMC9922720

[50] Redenšek S Dolžan V Kunej T. From genomics to omics landscapes of Parkinson’s disease: revealing the molecular mechanisms. OMICS. (2018) 22:1–16. doi: 10.1089/omi.2017.0181, PMID: 29356624 PMC5784788

[51] Zagare A Preciat G Nickels SL Luo X Monzel AS Gomez-Giro G. Omics data integration suggests a potential idiopathic Parkinson’s disease signature. Commun Biol. (2023) 6:1179. doi: 10.1038/s42003-023-05548-w, PMID: 37985891 PMC10662437

[52] Arafah A Khatoon S Rasool I Khan A Rather MA Abujabal KA. The future of precision medicine in the cure of Alzheimer’s disease. Biomedicines. (2023) 11:335. doi: 10.3390/biomedicines11020335, PMID: 36830872 PMC9953731

